# Crystallinity Engineering of Hematite Nanorods for High‐Efficiency Photoelectrochemical Water Splitting

**DOI:** 10.1002/advs.201500005

**Published:** 2015-03-16

**Authors:** Degao Wang, Yuying Zhang, Cheng Peng, Jianqiang Wang, Qing Huang, Shao Su, Lianhui Wang, Wei Huang, Chunhai Fan

**Affiliations:** ^1^ Division of Physical Biology, and Bioimaging CenterShanghai Synchrotron Radiation FacilityCAS Key Laboratory of Interfacial Physics and TechnologyShanghai Institute of Applied PhysicsChinese Academy of SciencesShanghai201800China; ^2^ Key Laboratory for Organic Electronics and Information Displays (KLOEID)Institute of Advanced Materials (IAM), and School of Materials Science and EngineeringNanjing University of Posts and Telecommunications9 Wenyuan RoadNanjing210046China

**Keywords:** antimony‐doped tin oxide nanoparticles, conductive substrate modification, crystallinity engineering, hematite nanorod, photoelectrochemical

## Abstract

**An effective strategy to overcome the morphology evolution of hematite nanorods under high‐temperature activation** is presented, via tuning the crystallinity and sintering temperature by substrate modification. It is demonstrated that the as‐prepared doping‐free hematite nanorods with fine nanostructures obtain a significantly higher photocurrent density of 2.12 mA cm^−2^ at 1.23 V versus RHE, due to effective charge separation and transfer.

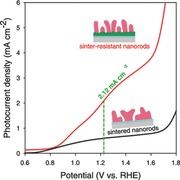

Photoelectrochemical (PEC) water splitting holds great promise for sustainable energy by harvesting solar energy. In particular, hematite (α‐Fe_2_O_3_) has been extensively investigated as a photo­anode for visible light‐driven PEC water splitting due to its availability, stability, and suitable bandgap of 1.9–2.2 eV.^1^, ^2^, ^3^ Fabricating hematite nanostructures provides an effective way to enhance the PEC performance of hematite photoanodes since they can enhance charge separation, surface area, and light absorption.^4^, ^5^, ^6^ High‐temperature activation (HTA) is a common method to eliminate the poor lattice mismatching between hematite and fluorine‐doped tin oxide (FTO) substrate.^7^, ^8^, ^9^ However, such hematite nanostructures often suffer from severe morphology evolution under HTA, resulting in the loss of fine structures that are critical for high‐performance PEC.^7^, ^10^, ^11^

High‐temperature annealing at ≈800 °C is a key step to activate hematite nanostructures for high‐performance PEC.^8^, ^10^ For instance, annealing at 800 °C improved the photocurrent density of hematite nanorods from 0.035 to 1.24 mA cm^−1^ at 1.23 V versus reversible hydrogen electrode (RHE), which represents one of the highest photocurrents reported for under hematite photoanodes under standard Air Mass (AM) 1.5 illumination.^8^, ^12^, ^13^ Nevertheless, the PEC performance of hematite nanostructures after HTA is still far below the theoretical expectation, partially because of morphology evolution during high‐temperature treatment. Such morphology evolution, e.g., distortion and coalescence, largely increases feature sizes, and decreases surface areas.^7^ The former impedes charge separation and the latter slows charge transfer at the electrode/electrolyte interface, both of which are detrimental to PEC. Therefore, it is highly desirable to fabricate hematite nanostructures with minimal morphology evolution after annealing at 800 °C for further improving the PEC water splitting efficiency. However, there has been few reports in this direction with the exception of a silica‐encapsulation technique.^11^ Whereas this strategy nicely controlled the morphology of porous hematite photoanode upon annealing at 800 °C, it suffered from considerable encapsulation‐induced anodic shift of the onset potential.

In this work, we have developed a novel strategy to rationally retain the morphology of hematite nanorods at high temperature and improve the performance of PEC water splitting. Our rationale stemmed from the thermodynamics of activation and sintering. Previous studies showed that the energy barrier associated with hematite activation is greater than that for morphology evolution.^10^ Hence, the temperature required for hematite activation is higher than the sintering temperature, resulting in significant morphology change. Given that the sintering temperature is closely related to the crystallinity of hematite,^14^, ^15^, ^16^ we reason that finely tuning the crystallinity of hematite nanostructures provides a feasible way to reduce lattice defects that are usually present in hematite nanostructures^17^ and increase their sintering temperature (**Figure** **1**). To achieve this, we employed commercially available antimony‐doped tin oxide (ATO) nanoparticles to decorate FTO substrate for the growth of vertically aligned hematite nanorods with high crystallinity. Such hematite nanorods largely retained their fine structures after HTA at 800 °C. As a result, we obtained photocurrent density of 2.12 mA cm^−2^ at 1.23 V versus RHE and 3.34 mA cm^−2^ at 1.50 V versus RHE, respectively, which represent the highest photocurrent density using unmodified hematite nanostructures.

**Figure 1:**
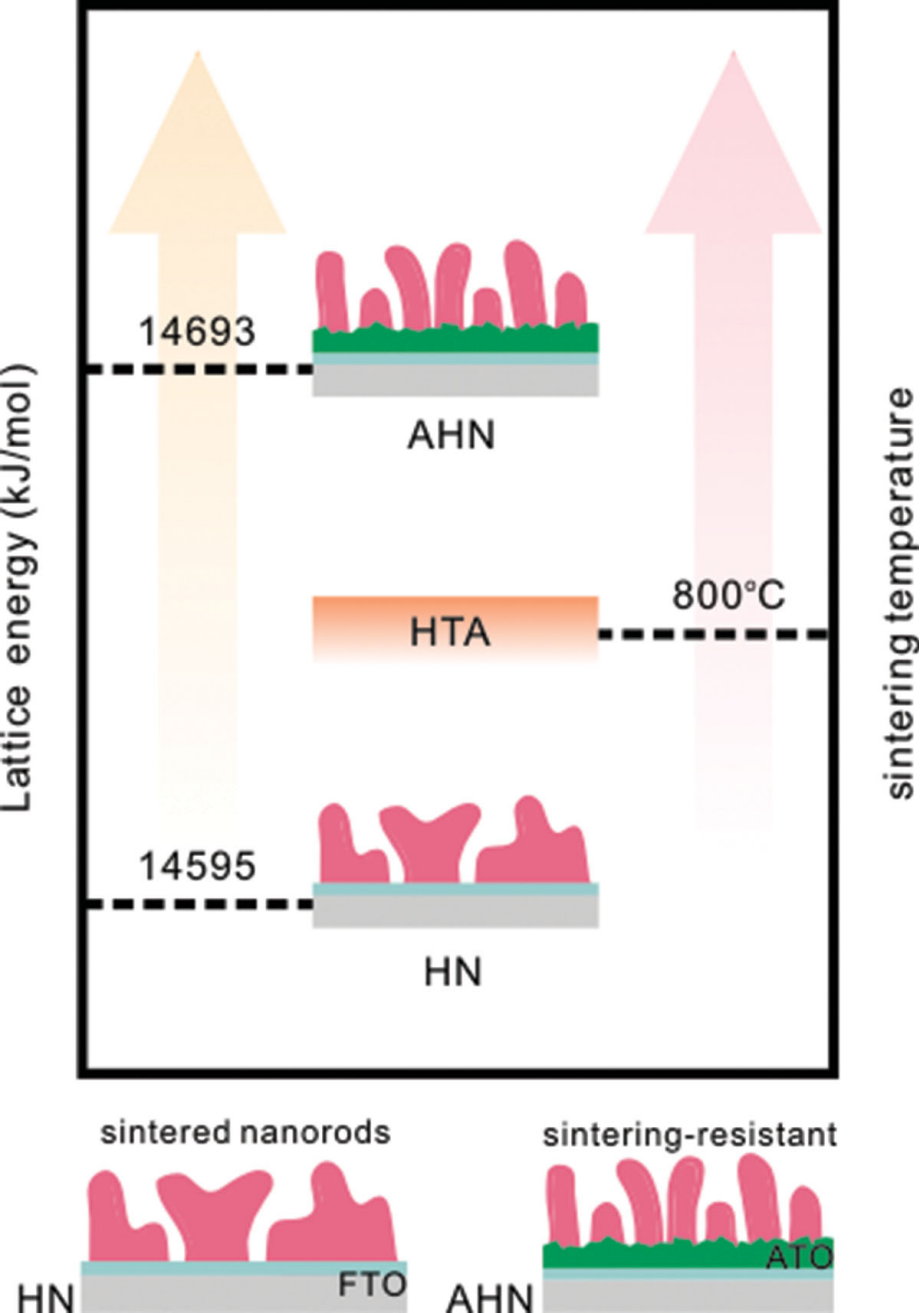
Energy and temperature diagram of both AHN and HN.

ATO decorated FTO conductive glass substrates (ATO–FTO) were fabricated by dropping ATO/ethanol colloidal dispersion on FTO substrates with further annealing at 500 °C. Vertically aligned akaganeite (β‐FeOOH) nanorods were then deposited on ATO–FTO substrates via the chemical bath deposition (CBD) process at 95 °C, and subsequently crystal phase was transited to hematite nanorods by annealing at 550 °C. To obtain high photoactive photoanodes, the as‐prepared hematite nanorods were activated by HTA at 800 °C (hematite nanorods on ATO, dubbed AHN). As a control, photoactive hematite nanorods on pristine FTO substrate were prepared with the same process (hematite nanorods on FTO, dubbed HN).

We characterized the morphology of hematite nanorods on both AHN and HN by scanning electron microscopy (SEM). As shown in **Figure** **2**b, most hematite nanorods on HN were distorted and coalesced, after HTA at 800 ^o^C, with characteristic sizes greater than 100 nm. The cross sectional SEM image further revealed that the bottom of hematite nanorods was coalesced whereas the top retained the rod‐like shape (Figure 2a). Hence, the morphology evolution suggests that hematite nanorods on HN after annealing are sintered at 800 °C. Remarkably, hematite nanorods on AHN largely retained their rod‐like shape on ATO–FTO (Figure 2e) after annealing at 800 °C. The interfaces are distinct at the bottoms of hematite nanorods (Figure 1f), indicating that hematite nanorods on AHN are sintering‐resistant during annealing at 800 °C. Furthermore, we found the main diameter of nonannealed hematite nanorods on AHN is smaller than that of ≈45 nm on HN (Figure S1, Supporting Information), suggesting that ATO modification influenced the crystal growth of hematite nanorods.

**Figure 2:**
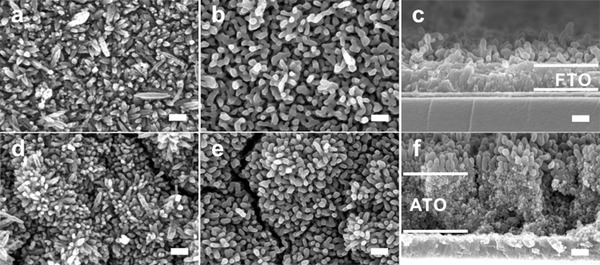
Morphology of AHN and HN before and after HTA at 800 °C. a) The SEM image of HN before HTA at 550 °C. b,c) The SEM image of HN after HTA at 800 °C. d) The SEM image of AHN before HTA at 800 °C. e,f) The SEM image of AHN after HTA at 800 °C. The scale bar in all images is 200 nm.

We further collected X‐ray diffraction (XRD) spectra to investigate the crystal structure of hematite nanorods on both AHN and HN. The XRD patterns (**Figure** **3**a) revealed that ATO modification tremendously intensified the (101) and (110) peaks of cassiterite phase and hematite nanorods with the intensified (012) and (014) faces grew on ATO–FTO. It indicates that ATO modification significantly influences the crystal structure of hematite nanorods. The lattice parameters calculated by MAUD software^18^, ^19^, ^20^ demonstrate that the lattice parameter of nanorods on AHN is closer to JCPDS card 33‐0664 (*a* = *b* = 5.0356, *c* = 13.7489) (Table S1, Supporting Information), suggesting the crystallinity of nanorods on AHN is higher than that on HN. Furthermore, the lattice energy was evaluated by Kapustinskii equation^21^ to quantitatively analyze the crystallinity. Given that the higher lattice energy indicates the higher crystallinity, the higher lattice energy of hematite nanorods on ATO–FTO confirms the crystallinity of AHN is higher than that of HN, as shown in Table S1, Supporting Information. Considering that higher lattice energy suggests higher temperature requirement for sintering, it further suggests that the sintering temperature of hematite nanorods on ATO–FTO is higher than that on FTO.

**Figure 3:**
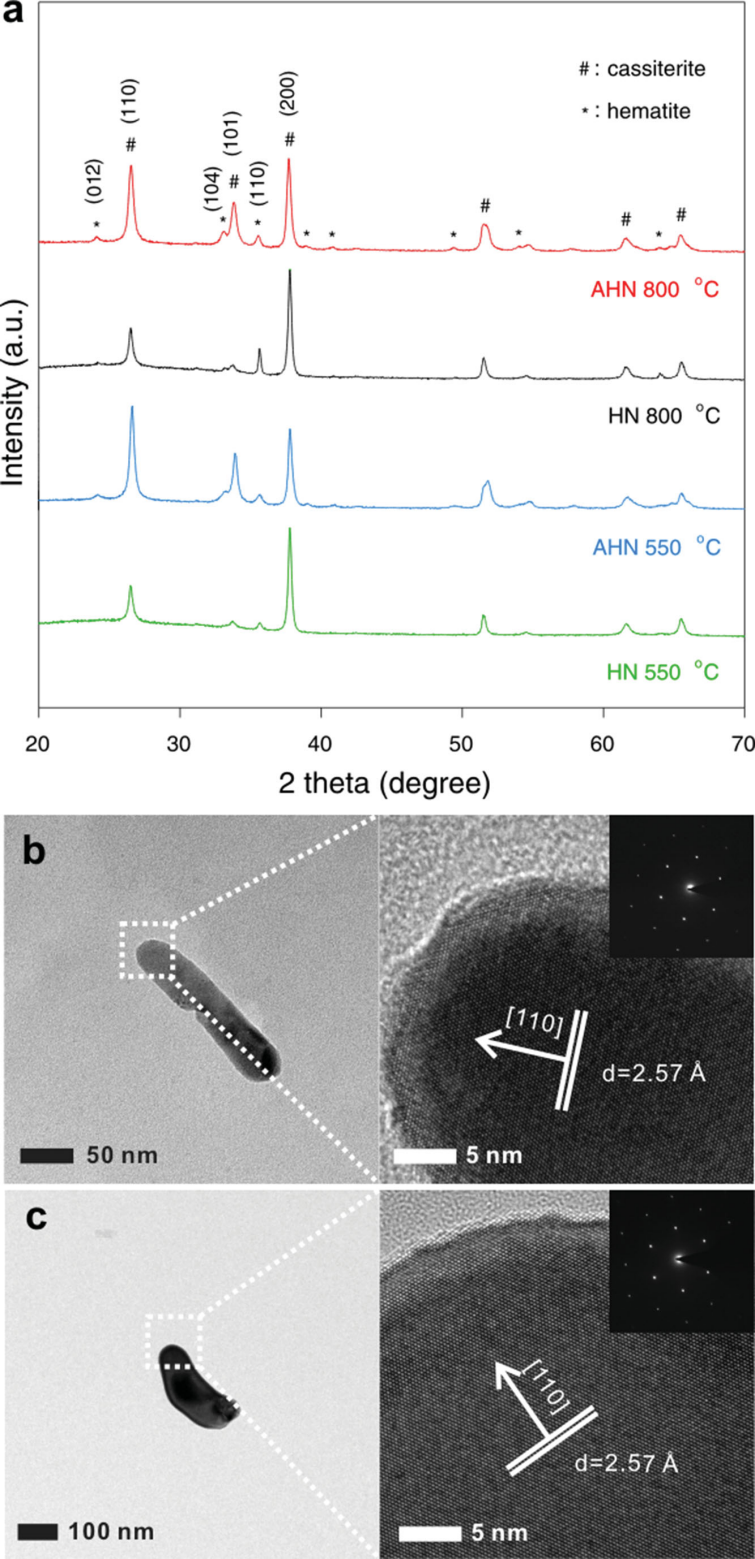
Structures of AHN and HN. a) The XRD patterns of AHN and HN before and after annealing at 800 °C. b) HRTEM images of AHN. c) HRTEM images of HN.

Of our particular interest is that the intensity of (110) peak in hematite phase, which directly reflects the sintering temperature. Figure 3a shows the (110) diffraction peak of HN is significantly enhanced to the strongest peak after annealing at 800 °C, which is attributed to the recrystallization of sintering. However, the (110) diffraction peak in AHN is slightly enhanced after annealing at 800 °C compared with HN, suggesting the absence of recrystallization or sinter. It confirms that the sintering temperature of hematite nanorods on HN is lower than 800 °C, whereas that of AHN is higher than 800 °C. Moreover, the strongest (110) diffraction peak suggests these sintered nanorods on HN prefer growing in the [110] direction,^22^, ^23^ as shown in the high resolution transmission electron microscopy (HRTEM) images in Figure 3c. The HRTEM images of nanorods on AHN after annealing at 800 °C are shown in Figure 3b. Of note, the (110) interplanar spacing of both AHN and HN are 2.57 Å, slightly larger than 2.52 Å in JCPDS card 330664. It should be attributed to the migration of Sn into the lattice of hematite driven by HTA.^8^, ^10^

**Figure** **4**a compares plots of photocurrent density versus potential for AHN and HN under simulated sunlight irradiation (100 mW cm^−2^, AM 1.5G). Upon irradiation, AHN yielded a photocurrent density of AHN reached 2.12 mA cm^−2^ at 1.23 V versus RHE, ≈3.5 times higher than that of HN (0.60 mA cm^−2^). Moreover, the photocurrent density reached 3.34 mA cm^−2^ at 1.50 V versus RHE before current density increased exponentially. To the best of our knowledge, this is the highest photocurrent density ever achieved using hematite nanorods without further doping, surface passivation or cocatalyst treatment. On the other hand, the onset potential of both AHN and HN are of ≈0.8 V versus RHE, suggesting the absence of onset potential anodic shift on AHN. It is consistent with the typical values ranging from 0.8 to 1.0 V observed in other hematite structures without catalyst.^8^, ^24^

**Figure 4:**
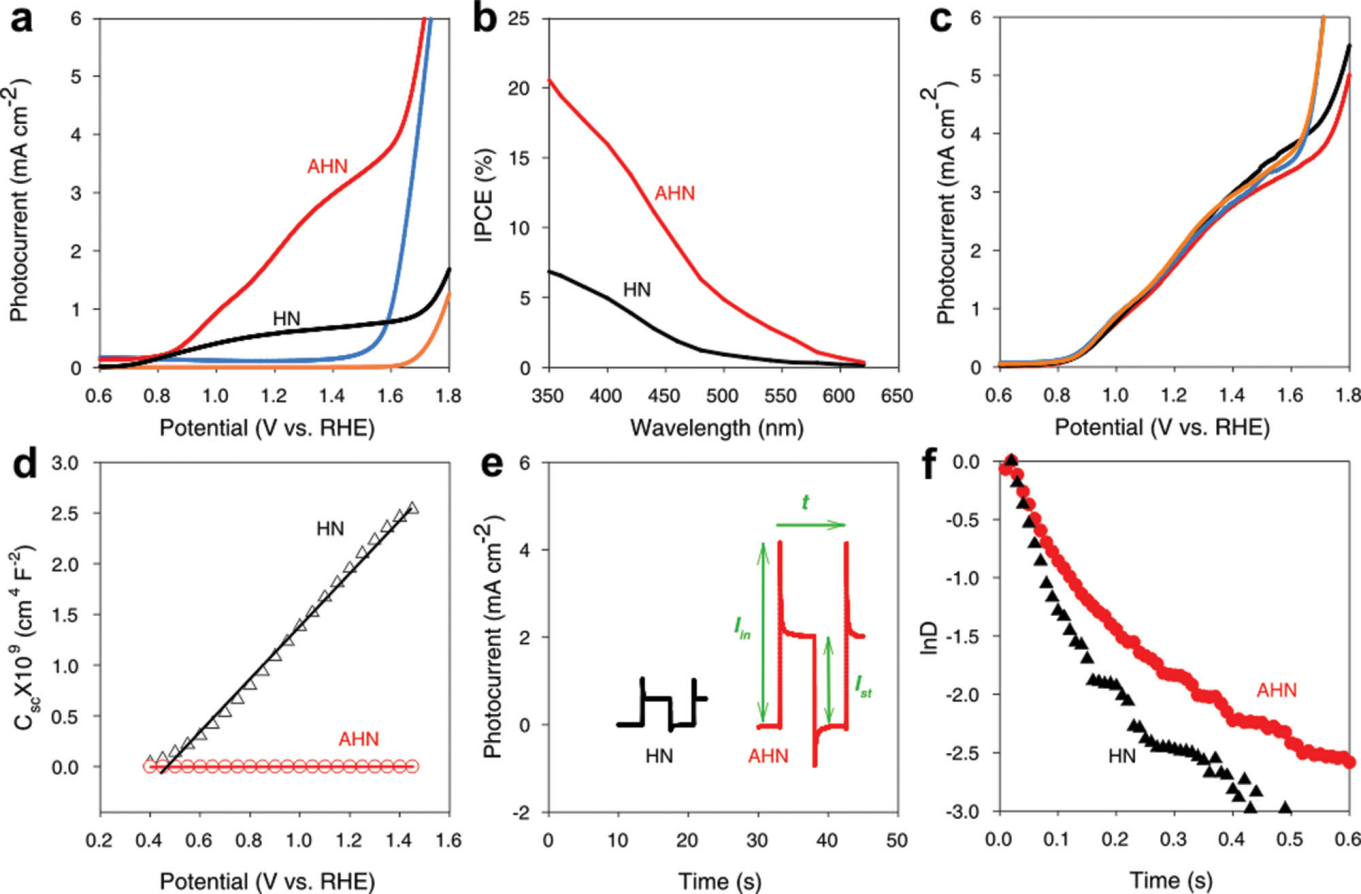
PEC performance of AHN and HN. a) ***J***–***V*** curves under the illumination of AM 1.5G full‐spectrum solar light with a power density of 100 mW cm^−2^. b) IPCE spectra at 1.23 V versus RHE. c) ***J***–***V*** curves of AHN with different thickness of ATO modification. d) Mott–Schottky plots. e) Transient photocurrent decay at the potential of 1.23 V versus RHE under illumination. f) Transient decay times.

Photocurrent densities of AHN with different amount of ATO modification were measured under the same condition. The similar *J*–*V* curves in Figure 4c strongly suggest that the photoresponse of AHN is independent to the amount of ATO modification, which is attributed to the excellent visible light transparency of ATO nanoparticles.^25^ In addition, we measured the bandgap of AHN and HN using UV−vis spectra and Tauc‐plot curves, respectively. As shown in Figure S2, Supporting Information, the bandgaps of both AHN and HN are ≈2.05 eV, suggesting the similar strong direct transition which is well consistent with other report.^7^

The incident‐photon‐to‐current efficiency (IPCE) spectra were then generated at 1.23 V versus RHE to compare the wavelength‐dependent photocurrent response for AHN and HN (Figure 4b). AHN exhibits substantially enhanced IPCE values as compared to HN over the entire wavelength of 350–620 nm. The maximum IPCE value of the AHN reached 20.6% at the wavelength of 350 nm, which is approximately three times higher than of that of HN (6.78%). The IPCE values of both AHN and HN gradually drop to zero at wavelengths longer than 600 nm, in accordance with the bandgap of hematite.

To understand the mechanism of the substantial enhancement in our PEC, we investigated the charge separation and transfer in the PEC process. Charge separation was evaluated by the density and lifetime of photo‐excited carriers in hematite nanorods. Mott–Schottky plots (Figure 4d) generated from electrochemical impedance spectroscopy show that the carrier density in hematite nanorods on AHN is 2.58 × 10^22^ cm^−3^, which is ≈5 orders of magnitude higher than that of HN (2.71 × 10^17^ cm^−3^). Photocurrent transient measurement (Figure 4e) was performed to quantitatively assess the carrier lifetime. The normalized *lnD*–*t* plots (Figure 4f) were then deduced from the photocurrent transient curves (Figure 4e) to calculate the transient time constant *τ*, a key factor defined as the time when *lnD* is −1.^26^ As shown in Figure 4f, *τ* was calculated to be 0.12 s for AHN, two times longer than 0.061 s of HN. The larger carrier density and longer carrier lifetime strongly imply higher charge separation efficiency of nanorods on AHN as compared to that on HN, which is due to the well‐retained morphology after sintering.

Morphology evolution during HTA seriously impedes the PEC performance using hematite nanostructures. While there have been several ways to address this problem by using external treatment,^11^ e.g., silica‐encapsulating technique, they often shift the onset potential to the anodic. In this study, we developed an “intrinsic” strategy of crystallinity engineering to raise the sintering temperature for the resistance of morphology evolution without introducing new materials on hematite nanorods or anodic shifting of the onset potential. By using this new strategy, we obtained the sintering‐resistant hematite nanorods with the water splitting photocurrent density of 2.12 mA cm^−2^ at 1.23 V versus RHE, representing the highest photocurrent density ever achieved by hematite nanorod‐based photoanodes without intentional dopants.

Given that substrate significantly influences the nucleation and growth of hematite nanorods in PEC process,^27^, ^28^, ^29^ we employ ATO modification to change the surface of FTO substrate (Figure S3, Supporting Information) to tune the crystal structure of hematite nanorods. Of particular importance, nanorods on AHN possess enhanced crystallinity, which significantly increases the sinter temperature as compared to that on HN. As a result, the morphology of nanorods on AHN is well‐retained after HTA at 800 °C, exhibiting distinct features including shape and size.

Compared with the sintered nanorods on HN, the sintering‐resistant nanorods on AHN have smaller diameter size and larger surface area, which are beneficial for the PEC performance due to the charge separation and transfer at the electrode/electrolyte interface. In a typical PEC process, the depletion layer is formed on the surface when hematite nanorods are placed in the electrolyte. Inside the depletion layer (Figure S4, Supporting Information), the core of hematite nanorod can be regarded as the diffusion region, where the electron–hole pairs are most likely lost via recombination due to the very short electron and hole diffusion length. Hence, photo‐excited electrons and holes are more effectively separated by electric field in the depletion layer than in the diffusion region.^1^, ^30^ Because of the morphology evolution after HTA, the feature size of nanorods on HN is significantly larger than that of the sintering‐resistant hematite nanorods on AHN. It suggests that the diffusion region in HN is larger than that in AHN, thus the contribution of depletion layer in AHN is larger than that in HN, and the charge separation efficiency in AHN is higher than that in HN. Also, the large surface area of hematite nanorods on AHN facilitates charge transfer for water oxidation. Moreover, the charge transfer resistance at the electrode/electrolyte interface on AHN is smaller than that on HN, indicating that the energy barrier for charge transfer across the electrode/electrolyte interface on AHN is lower than that on HN. Collectively, the efficiency of charge separation and transfer at the electrode/electrolyte interface of AHN is significantly higher than that of HN.

In summary, we have developed a crystallinity engineering strategy to effectively retain the morphology of hematite nanorods under HTA. Hematite nanorods growing on ATO–FTO have higher lattice energy and better sintering‐resistance, which overcomes the problem of morphology evolution during HTA. Importantly, as‐prepared hematite nanorods with fine nanostructures significantly improve the charge separation and transfer. As a result, the photocurrent density reached 2.12 mA cm^−2^ at 1.23 V versus RHE. Therefore, our work provides new insight into the morphology retainment of hematite nanostructures during HTA. Due to the use of commercially available ATO, this approach is also readily scalable and applied in a broad range of areas including dye‐sensitized solar cells, water electrolysis and supercapacitors.

## Supporting information

As a service to our authors and readers, this journal provides supporting information supplied by the authors. Such materials are peer reviewed and may be re‐organized for online delivery, but are not copy‐edited or typeset. Technical support issues arising from supporting information (other than missing files) should be addressed to the authors.

Supplementary
