# Supplementary material for: Crystallinity Engineering of Hematite Nanorods for High‐Efficiency Photoelectrochemical Water Splitting
Source: Adv Sci (Weinh). 2015 Mar 16;2(4):1500005. doi: 10.1002/advs.201500005 (PMC5016786; doi:10.1002/advs.201500005)
Supplement: Supplementary file 1 — Supplementary [file ADVS-2-0k-s001.pdf]

Copyright WILEY-VCH Verlag GmbH & Co. KGaA, 69469 Weinheim, Germany, 2015.

Supporting Information

for *Adv. Sci.*, DOI: 10.1002/advs.201500005

Crystallinity Engineering of Hematite Nanorods for High-Efficiency Photoelectrochemical Water Splitting

Degao Wang, Yuying Zhang, Cheng Peng,* Jianqiang Wang, Qing Huang, Shao Su,Lianhui Wang, Wei Huang, and Chunhai Fan*

Copyright WILEY-VCH Verlag GmbH & Co. KGaA, 69469 Weinheim, Germany, 2013.

Supporting Information

Crystallinity engineering of hematite nanorods for high-efficiency photoelectrochemical water splitting

Degao Wang, Yuying Zhang, Cheng Peng, Jianqiang Wang, Qing Huang, Shao Su, Lianhui Wang, Wei Huang, and Chunhai Fan**

Experimental section.
Materials: Ferric chloride ($FeCl_3$•$6H_2O$), $NaNO_3$ and acetone, anhydrous ethanol were all A.R. grade and purchased from Sinopharm Chemical Reagent Co. Ltd. at Shanghai. All aqueous solutions were prepared with deionized (DI) water from Milli-Q-Water (Millipore Corp, 18.2 MΩ/cm at 25 ^{o}C). Fluorine-doped tin oxide coated glasses (FTO, TEC-15) were purchased from NGS glass, and each piece of FTO glass was cleaned ultrasonically in acetone, pure water and ethanol, respectively. Antimony-doped tin oxide (ATO) nanoparticles with the average particle size of 13-22 nm was purchased from Alfa Aesar. The weight ratio of Sb_2O_5/SnO_2 is 15:85 and the specific surface area of ATO particles is 42-72 m^2/g.
ATO modification: ATO modified FTO (ATO-FTO) conductive substrates was prepared by dropping the ATO/ethanol dispersions (50 μg/mL) on the FTO glass with an annealing treatment at 500 ^{o}C for 1 h in the air condition.[1-3] Then, the vertically aligned β-FeOOH nanorods on the ATO-FTO conductive substrate were prepared via chemical bath deposition (CBD),[4, 5] and subsequently the hematite nanorods were formed after annealing treatment at 550 ^{o}C for 2 h and 800 ^oC for 14 min, respectively. TEM samples were prepared by scraping the hematite nanorods grown on the ATO/FTO and FTO substrates with a razor blade. The hematite was transferred to a little vial with 1mL ethanol that was placed in a bath sonicator for 20 seconds, followed by pipetting the mixture onto a carbon-coated TEM grid. ATO/ethanol dispersions with different concentration (including 25, 50, 75 and 100 μg/mL) were used to adjust the thickness of ATO layer, and the ***J-V*** curves of these AHN photoanodes were collected under the same condition.
Characterization: The morphologies of all samples were characterized by scanning electron microscope (SEM, JME2011, JEOL, Japan) and high-resolution transmission electron microscope (HRTEM, FEI TECNAI G^2 F20). X-ray diffraction spectra (XRD) were carried out with X-ray diffractometer (Bruker AXS, D8 Advanced) using Cu Kα radiation (λ=1.5418 Å). Electrochemical impedance spectra (EIS) were collected by Autolab electrochemical workstation (Metrohm AG, Switzerland).
PEC Measurements: The vertically aligned hematite-nanorod photoanode with a bare portion of FTO substrate was fabricated by soldering with copper wire and all samples were sealed on all edges with epoxy resin except a bare area of 0.2 cm^2 confined by the epoxy for photo excitation. All photoelectrochemical measurements were tested in a three electrode configuration with the Pt counter electrode, the Ag/AgCl reference electrode (BASi) and the working electrode of the photoanode. An aqueous solution of 1.0 M NaOH (pH 13.6) after deaerating with a nitrogen flow was filled in a quartz PEC cell as the electrolyte. A solar simulator (Newport, Model SP 94023A) coupled to a filter (AM 1.5G) using 150 W Xenon lamp was used as the light source. The light power density of 1000 W/m^2 was measured with a power meter (Newport, 91150V). Incident-photon-to-current efficiency (IPCE) spectra were

measured at 1.23 V vs. RHE as a function of the wavelength of the incident light by an electrochemical station (CHI 650b) with a solar simulator (Newport 66902, 500 W xenon lamp), coupled to an aligned monochromator (Newport 74125) and a Si detector (Newport 71675). The exposed area on the samples was confined by epoxy resin at 0.2 cm^2, which is far greater than the spot size (A_{ph}= 0.1 mm × 0.2 mm) of monochromated light. Thus the photocurrent was calculated using the equation below:

$j_{ph} = \frac{I_{ph}-I_d}{A_{ph}}$ $j_{ph} = \frac{I_{ph}-I_d}{A_{ph}}$ (1)

where I_{ph} and I_d was the current tested under chromated light illumination and in the dark, respectively. The intensity of the monochromated light was calibrated using a Si photodetector at zero bias. Thus the IPCE was calculated by using the equation below:[6]

$j_{ph} = \frac{I_{ph}-I_d}{A_{ph}}$ (2)

Where j_{phD} was the photocurrent from the Si photodetector in mA/cm^2, and η_{ext} was the external quantum efficiency of the Si photodetector.

The electrochemical AC impedance spectroscopy were performed in the dark configuration system in 1.0 M NaOH solution with a sinusoidal perturbation with 50 mV amplitude and frequencies ranging from 100 kHz to 1 Hz. Capacitance values were derived from the impedance-potential and Mott-Schottky plots were generated from the capacitance values.

The collected potentials vs. Ag/AgCl were converted to the reversible hydrogen electrode (RHE) scale according to the Nernst equation:

$E_{RHE} = E_{Ag/AgCl} + 0.059pH + E^0_{Ag/AgCl}$ (3)

where E_{RHE} was the converted potential vs. RHE, $E^0_{Ag/AgCl}$ was 0.1976 V at 25 °C, and $E_{Ag/AgCl}$ was the experimental potential against the Ag/AgCl reference. Since the solution pH value was 13.6, the $E_{Ag/AgCl}$ could be converted to E_{RHE} according to the following equation:

$E_{RHE} = E_{Ag/AgCl} + 1$ (4)

The carrier densities were calculated from the slope of Mott-Schottky plot by the following equation:

$(\frac{1}{C})^2 = (\frac{2}{e_0 \varepsilon_0 \varepsilon N_d})(E - E_{FB} - \frac{kT}{e_0})$ (5)

where C was capacitance (F/cm^2), e_0 was the electron charge, ε was the hematite dielectric constant of 80, ε_0 was the permittivity of vacuum, N_d was the carrier density, E was the electrode applied potential, E_{FB} was the flatband potential, k was the Boltzmann's constant and T was the temperature.

References

[1] D. G. Wang, Y. Y. Zhang, J. Q. Wang, C. Peng, Q. Huang, S. Su, L. H. Wang, W. Huang, C. H. Fan, *ACS Appl. Mater. Interfaces* **2014**, *6*, 36.

[2] J. Moir, N. Soheilnia, P. O'Brien, A. Jelle, C. M. Grozea, D. Faulkner, M. G. Helander, G. A. Ozin, *ACS Nano* **2013**, *7*, 4261.

[3] Q. Peng, B. Kalanyan, P. Hoertz, A. Miller, D. H. Kim, K. Hanson, L. Alibabaei, J. Liu, T. J. Meyer, G. N. Parsons, J. T. Glass, *Nano Lett.*, **2013**, *13*, 1481–1488.

[4] P. Wang, D. G. Wang, J. Lin, X. L. Li, C. Peng, X. Y. Gao, Q. Huang, J. Q. Wang, H. J. Xu, C. H. Fan, *ACS Appl. Mater. Interfaces* **2012**, *4*, 2295.

[5] L. Vayssieres, A. Hagfeldt, S. E. Lindquist, *Pure Appl. Chem.* **2000**, *72*, 47.

[6] K. Sun, X. L. Pang, S. H. Shen, X. Q. Qian, J. S. Cheung, D. L. Wang, *Nano Lett.* **2013**, *13*, 2064.

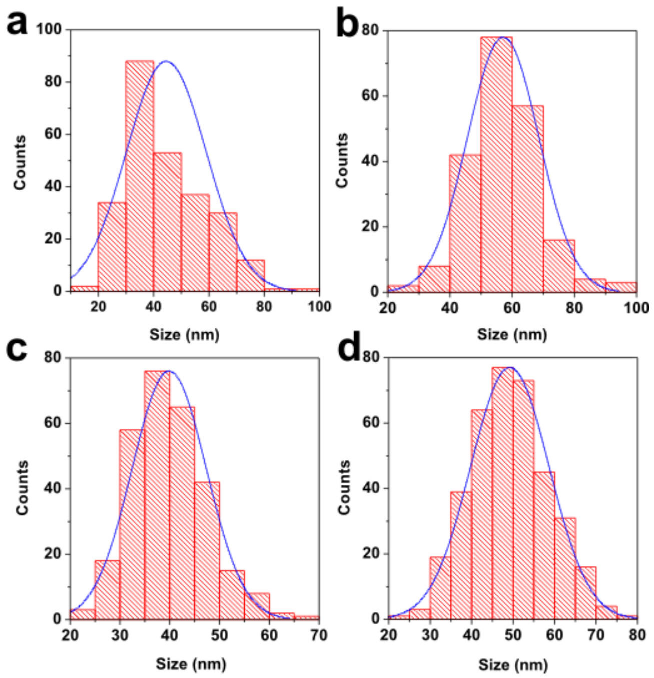

Figure S1. The main diameter distribution of HN (a, b) and AHN (c, d) before and after HTA at 800 ^{o}C.

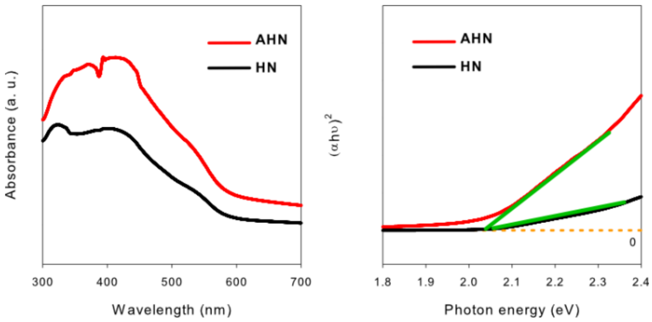

Figure S2. UV-vis absorption spectra (a) and Tauc plots for the indirect case (b) and the direct case (c) of HN and AHN.

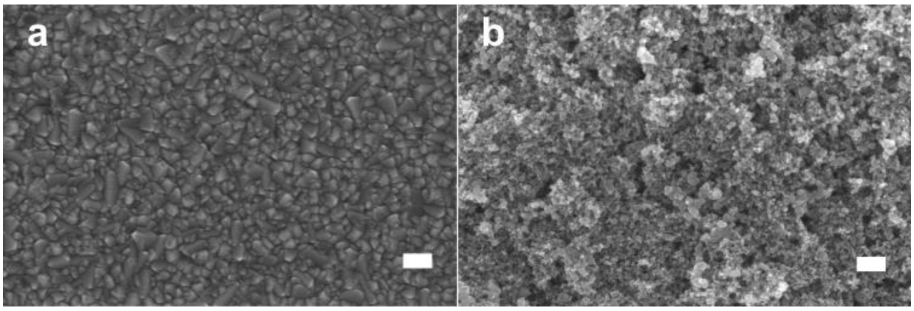

Figure S3. The SEM image of ATO modified FTO substrate (a) and bare FTO substrate (b), the scale bar is 200 nm.

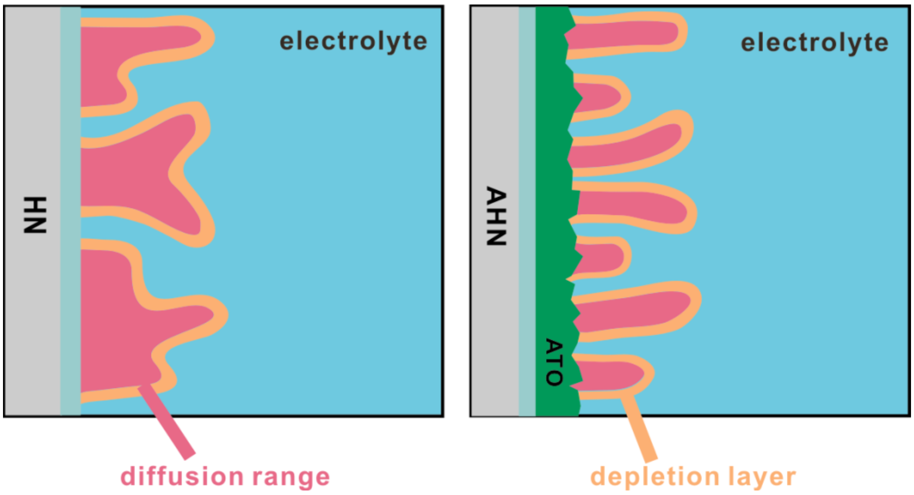

Figure S4. Illumination of charge separation and transport at the electrode/electrolyte interface of AHN and HN, respectively.

Table S1. Lattice parameters of non-annealed hematite nanorods on AHN and HN.

	**lattice parameters (Å)**		**Fe-O band distances (Å)**		**Lattice energy (kJ mol^{-1})**	**Refinement reliability parameters**	
	a=b	c	short band	long bond		Sig	Rw %
HN 550 ^{o}C	5.1110	13.9089	1.9724	2.1419	14595.41	1.30	3.85
AHN 550 ^{o}C	5.0434	13.7616	1.9210	2.1588	14693.35	1.37	4.20
